# Longitudinal neural and psychological changes following psilocybin under compassion imagery priming

**DOI:** 10.3389/fnhum.2026.1874178

**Published:** 2026-07-22

**Authors:** Carla Pallavicini, Lorena Llobenes, Federico Cavanna, Laura Alethia de la Fuente, Stephanie Muller, María Costa, Natali Gumiy, Nicolás Bruno, Tomás D'Amelio, Jaskaran Basran, Ptarmigan Plowright, Federico Zamberlan, Guillermo Pasqualetti, Ain Stolkiner, Mauro Namías, Paul Gilbert, Enzo Tagliazucchi

**Affiliations:** 1 Department of Physics, Institute of Applied and Interdisciplinary Physics, University of Buenos Aires, Buenos Aires, Argentina; 2 National Scientific and Technical Research Council, Buenos Aires, Argentina; 3 Cognitive Neuroanatomy Lab, The Integrative Neuroscience and Cognition Center (INCC UMR 8002), CNRS, Université Paris Cité, Paris, France; 4 Motivación Compasiva, Buenos Aires, Argentina; 5 Institute of Cognitive and Translational Neuroscience, The Institute of Cognitive and Translational Neuroscience (INECO) Foundation, Favaloro University, Buenos Aires, Argentina; 6 Uruguayan Center for Molecular Imaging, CUDIM, Montevideo, Uruguay; 7 Institut du Cerveau—Paris Brain Institute—ICM, Sorbonne Université, Paris, France; 8 Centre de Recerca Matemàtica (CRM), Universitat Autónoma de Barcelona, Bellaterra, Barcelona, Spain; 9 Centre for Compassion Research and Training, College of Science and Engineering University of Derby, Derby, United Kingdom; 10 The Compassionate Mind Foundation, Derby, United Kingdom; 11 Department of Cognitive Science and Artificial Intelligence, Tilburg University, Tilburg, North Brabant, Netherlands; 12 Fundación Centro Diagnóstico Nuclear, Buenos Aires, Argentina; 13 Cognitive Neuroscience Center, Universidad de San Andrés, Buenos Aires, Argentina; 14 Latin American Brain Health Institute (BrainLat), Universidad Adolfo Ibanez, Santiago, Chile

**Keywords:** absorption, cognitive neuroscience, compassion imagery, fMRI, functional connectivity, psilocybin, resting-state networks, self-compassion

## Abstract

Psilocybin is a classic psychedelic known to alter subjective experience and induce lasting psychological changes, including increased cognitive flexibility and reduced rigid self-related beliefs. Contextual factors, including preparatory mental states, are thought to play a key role in shaping these effects. Compassion imagery, which engages care-affiliative motivational systems, may represent a promising priming approach in this context. Here, we investigated the neural and psychological effects of psilocybin under compassion imagery priming using self-report questionnaires and functional magnetic resonance imaging (fMRI) in a naturalistic sample of 105 participants. Participants were primed with either compassion imagery or attention to breathing prior to psilocybin intake. We observed sustained increases in absorption over time, particularly in the high-dose compassion imagery condition. Additional within-group increases were found in measures of decentering and self-compassion. Using functional connectivity features, fMRI-based classifiers distinguished between resting state and compassion imagery conditions prior to intake, and between priming conditions following psilocybin, with significant classification performance observed only in the high-dose group. These findings suggest that psilocybin is associated with lasting changes in absorption and may be modulated by contextual priming to shape both psychological outcomes and large-scale brain network dynamics. More generally, our results highlight the potential role of compassion-based practices as preparatory interventions in psychedelic contexts. Future confirmatory studies are needed to further characterize these effects and their underlying mechanisms.

## 1 Introduction

Psychedelic substances, once shrouded in stigma and controversy, have recently garnered renewed attention for their potential therapeutic benefits (Griffiths et al., 2016; Gründer and Jungaberle, 2021; Kim et al., 2025; Nichols and Walter, 2021; Perkins et al., 2021). Among these substances, psilocybin, found in certain species of mushrooms, has emerged as a promising candidate for mental health intervention (Griffiths et al., 2016; Goldberg et al., 2020; Carhart-Harris et al., 2021; Goodwin et al., 2022). Recent research has revealed compelling evidence of psilocybin's capacity to promote psychological wellbeing and induce enduring positive changes in personality traits (Bouso et al., 2015, 2018; Griffiths et al., 2011; MacLean et al., 2011; Nichols, 2016; Nichols et al., 2017; Perkins et al., 2021). These long-term modifications can be elicited by a single dose of psilocybin and are closely related to the quality of the subjective experience during the acute psychedelic state (Garcia-Romeu et al., 2014; Griffiths et al., 2008, 2011, 2016; MacLean et al., 2011; Millière et al., 2018). When generating altered states of consciousness, psilocybin can create peak experiences, also referred to as “mystical experiences”. These states are characterized by ego dissolution, forms of self-transcendence and inter-connectedness, feeling part of “all things”, oceanic boundlessness, and activation of positive affect sometimes referred to as awe and bliss (Griffiths et al., 2008; Tagliazucchi et al., 2016; Milliere, 2017; Pallavicini et al., 2021). These changes have been linked to enduring changes in personality, behavior, and wellbeing (Griffiths et al., 2011). Moreover, neuroimaging studies have demonstrated that psilocybin may facilitate the activation of the core areas of what has been called the “social brain” (Slavich et al., 2023) that evolved to facilitate attachment, social connectedness, friendship and cooperation (Rodríguez Arce and Winkelman, 2021). These brain areas are particularly sensitive to social information in regard to the similarity, support and helpfulness of others and impact social cognition, and social reward systems, potentially offering insights into the therapeutic mechanisms of psilocybin (Carhart-Harris et al., 2012; Preller and Vollenweider, 2018; Nardou et al., 2023). In addition, compassion and compassion training have also been shown to impact many areas of the social brain, for example the ventral striatum, the (subgenual) anterior cingulate cortex, and the orbitofrontal cortex, along with neural networks associated with positive emotion and emotion regulation (Förster and Kanske, 2022; Kim et al., 2020; Vrtička et al., 2017).

The Relaxed Beliefs Under pSychedelics (REBUS) model (Carhart-Harris and Friston, 2019) provides a theoretical account of the mechanisms through which psychedelics produce positive therapeutic outcomes. Grounded in the Bayesian predictive processing framework (Clark et al., 2018; Friston, 2010), REBUS proposes that psychedelics relax the precision-weighting of high-level priors (how strongly the brain relies on its top-down beliefs when interpreting reality), reducing the rigidity of maladaptive beliefs and increasing both neural and psychological flexibility. As with other facets of the psychedelic acute effects, this outcome is intricately tied to the context of the experience, also known as “set and setting”, the former referring to the brain state at the time of ingestion in regards to a sense of social safeness or unfamiliarity and threat, and the latter to the external environment (Hartogsohn, 2016; Studerus et al., 2012; Haijen et al., 2018; Carhart-Harris et al., 2018). While implementing settings conducive to the therapeutic action of psychedelics can be relatively straightforward, identifying and eliciting a favorable mindset remains more challenging, but in view of the processes involved in the evolution of the social brain (Slavich et al., 2023; Rodríguez Arce and Winkelman, 2021) and their psychophysiological mediators, it is likely to be linked to issues of social trust and care-protection. Herein lies the potential value of contemplative practices as primes because these practices stimulate core brain areas linked to feelings of social connectedness and caring (Förster and Kanske, 2022; Kim et al., 2020; Vrtička et al., 2017). These conditions have already shown promise in eliciting positive outcomes when combined with psilocybin and other classic serotonergic psychedelics (Griffiths et al., 2018; Millière et al., 2018; Heuschkel and Kuypers, 2020; Holas and Kamińska, 2023; Timmermann et al., 2023).

Compassion-Focused Therapy (CFT; Gilbert, 2000, 2014, 2020, 2022, 2023; Petrocchi et al., 2024) and Compassionate Mind Training (CMT) were developed to help individuals with high shame and self-criticism, who often respond poorly to traditional cognitive-behavioral interventions because of difficulties in generating self-soothing compassionate and supportive inner dialogue. Grounded in evolutionary psychology, attachment theory, and social mentality theory, CFT is a biopsychosocial therapy model that conceptualizes compassion as an evolved (stimulus-response guided) motivation, rooted in evolved caregiving systems. The evolution of caring helps the “cared-for” to regulate threat and seek social connectedness, affiliation and friendships as major evolved biosocial goals (Rodríguez Arce and Winkelman, 2021). Compassion, defined as a “sensitivity to suffering and needs in self and others, with a commitment to alleviate and prevent it” (Gilbert, 2014, 2022) therefore involves two parts: the stimulus which is the sensitivity to suffering and the response which is commitment to alleviating and preventing it. This motivation can operate through three flows: toward others, from others, and toward oneself; each supported by distinct psychophysiological mechanisms. These affiliative processes engage a balancing of the autonomic system with appropriate parasympathetic regulation via the vagus nerve, promoting states of safeness, affiliation, social trust, and flexible attention (Geller and Porges, 2014; Petrocchi and Cheli, 2019). Meta-analytic evidence across 47 studies indicates that CFT produces robust reductions in overall mental health difficulties across clinical and non-clinical populations, supporting its transdiagnostic efficacy (Petrocchi et al., 2024).

From a predictive processing perspective, both compassion training and psychedelics can be understood as processes that facilitate the updating of internal models (Clark et al., 2018; Friston, 2010; Carhart-Harris and Friston, 2019; Gilbert, 2020). Compassion reorients motivational systems from threat-sensitive, competitive strategies toward caring, prosocial motivations (Gilbert, 2014, 2022), thereby guiding the brain to update predictive models toward greater safeness and social connectedness. Psychedelics, by temporarily increasing cognitive flexibility and reducing the precision of rigid self-related beliefs, may potentiate this process (Carhart-Harris and Friston, 2019). Together, these approaches may shape subjective experience by increasing cognitive flexibility while engaging affiliative motivational systems (Millière et al., 2018; Timmermann et al., 2023).

In this framework, compassion imagery is conceptualized as a contextual priming manipulation rather than an independent intervention. To empirically test this, we designed a naturalistic field study combining psilocybin intake with compassion imagery (CI) or attention-to-breathing (AB) priming. Participants were randomly assigned to engage in one of these priming conditions prior to the psilocybin session. Compassionate imagery was used to activate caring-based motivational systems through the generation of affiliative and soothing emotions, while attention to breath served as a structurally similar contemplative task engaging attentional networks. The effects of psilocybin were examined by comparing a high dose (HD) and a low dose (LD) within a naturalistic setting. The experimental design assessed subjective effects and changes in self-reported scales, and incorporated functional magnetic resonance imaging (fMRI) to examine neural correlates, with a particular focus on whether compassion imagery priming was associated with changes in brain functional connectivity following psilocybin. Our main hypothesis was that psilocybin-related effects on psychological processes related to subjective wellbeing would vary as a function of compassion imagery priming, with corresponding changes in the functional connectivity of large-scale brain networks.

## 2 Methods

### 2.1 Overview of the experiment

**Figure 1:**
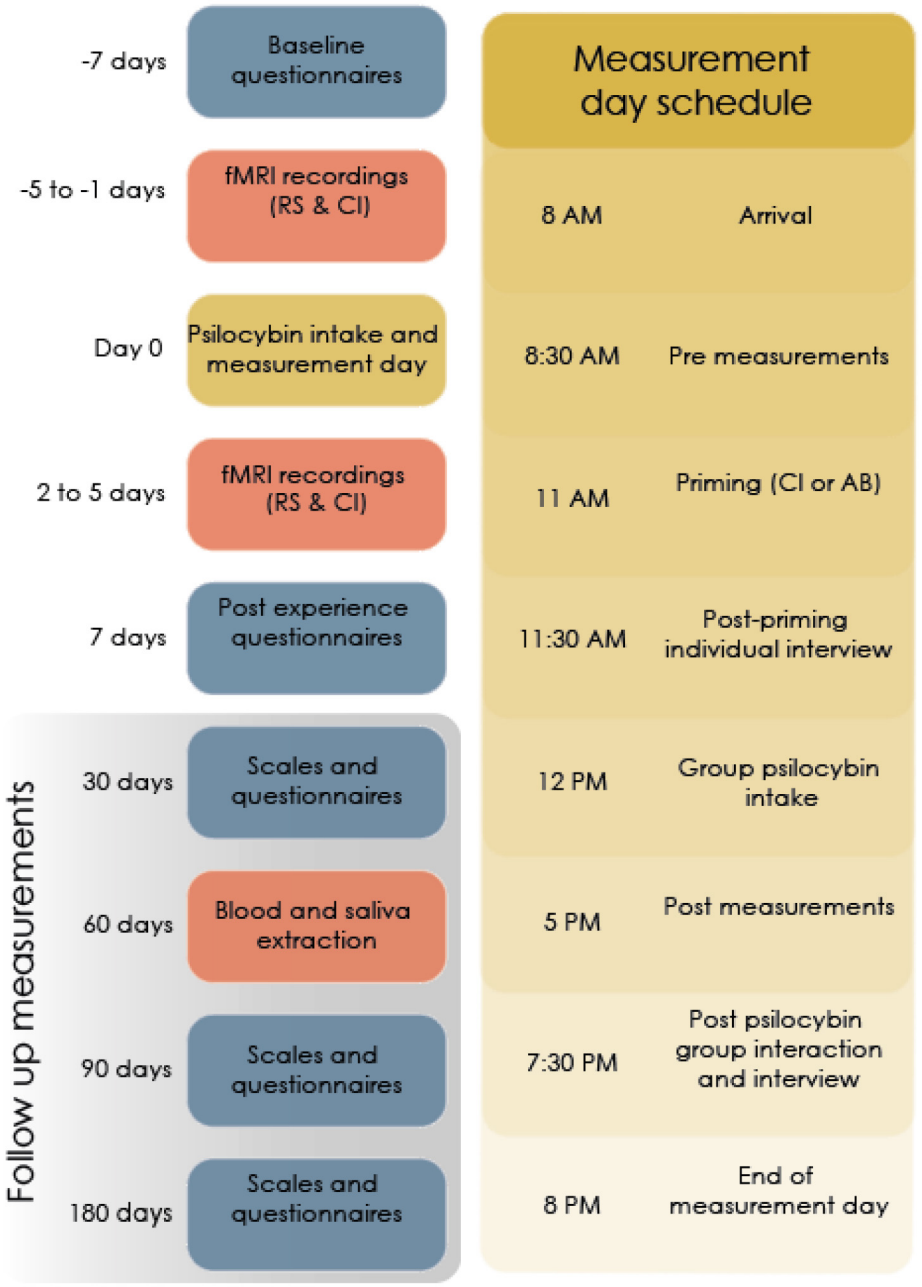
Overview of the experimental protocol. The left flow chart summarizes the questionnaires and biological/neuroimaging measurements performed before and after the psilocybin intake (day 0). The color of the boxes indicate the location of the measurements: remote (blue); medical center CDN (red); experience site (green). Neuroimaging acquisitions consisted of fMRI data acquired during resting state (RS) and during the practice of compassionate imagery (CI). Blood and saliva samples were obtained for analysis in a future report. The right flow chart presents the typical schedule of the psilocybin intake and measurement day. Before the group psilocybin intake, participants were briefly primed with either compassionate imagery or attention to breathing (AB).

The experimental procedure is illustrated in Figure 1. Participants were recruited and screened according to the procedures outlined in the “Participants” subsection. After assessment of the inclusion criteria, a personalized timeline was established around each participant's preferred intake day. One week prior to this day, participants completed questionnaires online and received Fitbit wristbands to record physical activity data, which they wore until 1 week after the experience. During the week preceding the intake day, participants visited the medical facility “Centro Diagnóstico Nuclear” (CDN) for fMRI scans (monitored for 5 min resting-state and 5 min compassionate imagery task) and provided blood and saliva samples. Primary outcomes focused on contemplative abilities, including compassion and absorption. Secondary measures, such as personality traits and additional psychological variables, were collected but will be analyzed in future work.

On the day of the psilocybin intake, groups of 5 to 10 participants arrived at the location at 8 am to complete pre-experience assessments, including scales, questionnaires, interviews, and EEG recordings [5 min resting-state (RS) and 5 min compassionate imagery task (CIT)]. Following this, participants underwent contemplative priming: Compassionate Imagery (CI) for active groups and Attention to Breathing (AB) for controls, lasting approximately 20 min. Individual interviews were then recorded. Subsequently, participants and their facilitator moved to a private area, with the time of psilocybin intake recorded. During the following 5 h there was no intervention or interaction with researchers unless requested by a participant. After this period, participants underwent post-experience measures, including saliva extraction, scales, questionnaires, EEG recordings (5 min resting-state and 5 min compassionate imagery), and individual and group interviews. Participants left the location around 8 pm.

During the week after the ceremony, participants returned to the medical facility for follow-up MRI scans and blood and saliva samples. Fitbit bands were returned, and participants completed questionnaires online 7 days after the ceremony. Follow-up measures included online questionnaires at 1, 3, and 6 months after the intake day, with a blood draw at 2 months post-experience.

The study was not preregistered. Given the naturalistic design and the broad range of behavioral and neuroimaging measures acquired, several analyses reported in the present work should be considered exploratory and hypothesis-generating.

### 2.2 Participants

Participants were recruited through word of mouth and social media advertising from October 2021 to June 2022. A screening process conducted by mental health professionals, involving the Depression Anxiety and Stress Scale−21 (DASS-21) (Antony et al., 1998; Lovibond and Lovibond, 1995) and a non-diagnostic interview was used to assess volunteers' suitability. During this initial screening, participants were briefed on the experiment's details, objectives, and inclusion/exclusion criteria, which are detailed below.

Participants who fulfilled inclusion criteria received an information sheet outlining the study procedures and provided written informed consent prior to participation. A total of 107 participants [52 female, 55 males ages 38 ± 8 years (mean ± std)] were recruited for the study. Two participants were unable to begin the experiment. As a result, 105 participants completed all assessments up to and including the psilocybin intake day. Five participants withdrew after the psilocybin intake day so 100 completed the post-intake fMRI session. Due to dropout, this was reduced to 93 participants at follow up. Participants were free to withdraw from the study at any point without providing justification, in accordance with ethical guidelines and the informed consent procedure. Reasons for withdrawal were not systematically recorded.

Participants had previously agreed with a facilitator to partake in a group experience with psilocybin and agreed to participate in the experimental protocol undergoing the measurements described in the previous subsection, which required blinding regarding the received dose of psilocybin. They were also informed that psilocybin would not be provided by the research team, and that the researchers would only conduct measurements before and after the acute effects.

The study was conducted in accordance with the Helsinki Declaration and approved by the Committee for Research Ethics at Universidad Abierta Interamericana University (Buenos Aires, Argentina) on May 31, 2021 (Reference Number: 0-1068).

### 2.3 Inclusion and exclusion criteria

To be included in the experiment, participants were required to have a previous arrangement with the facilitator with the intention of participating in a group psilocybin experience. They also agreed to be blinded regarding the received dose of psilocybin. Participants agreed to receive contemplative training (CI or AB), undergo non-diagnostic EEG and fMRI scans, a non-diagnostic interview with a mental health professional, blood and saliva extractions and to wear a FitBit wristband during 2 weeks. Participants included in the experiment also agreed to avoid consuming psychoactive drugs at least 1 week before the beginning of the experiment, and to abstain from tobacco, alcohol and caffeine consumption at least 24 h before their intake date.

All participants were aged between 21 and 65 years. Pregnant women were excluded from the experiment, as well as people with metallic material implanted in their bodies, people with pacemakers and claustrophobia. Participants who declared past difficult experiences with psychedelics with lasting negative psychological sequelae or who put themselves or others at risk were excluded from the experiment. A non-diagnostic psychiatric interview (SCID-CT; First, 2014) was conducted according to the guidelines by Johnson et al. (2008). Participants who fulfilled DSM-IV criteria for the following disorders were excluded from the experiment: schizophrenia or other psychotic disorders, and type 1 or 2 bipolar disorder (both also in first and second degree relatives), substance abuse or dependence over the last 5 years (excluding nicotine), depressive disorders, recurrent depressive episodes, obsessive-compulsive disorder, generalized anxiety disorder, dysthymia, panic disorder, bulimia or anorexia, as well as Participants with history of neurological disorders. Participants who presented one standard deviation above the mean in the State-Trait Anxiety Inventory (Spielberger, 2010) were excluded, as well as participants under psychiatric medication of any kind. All participants were required to be willing and able to provide informed consent.

### 2.4 Experimental conditions and psilocybin administration

Due to the naturalistic design, participants were enrolled on a rolling basis as they became eligible and completed screening procedures. Rather than being randomly assigned, participants selected among available ceremony dates according to their availability, and groups were subsequently formed around those dates. Consequently, the study was not designed to prospectively balance demographic or experiential variables across conditions. Once participants signed the informed consent, they were put in touch with the organizing team who received information concerning the planned psilocybin intake day. Participants chose their preferred date, and groups were arranged with the facilitator based on these preferences. As a control for psilocybin consumption and compassionate imagery priming, we used an active placebo consisting of a lower dose combined with a contemplative practice which only focused attention to breathing.

The 100 participants who completed the post-measurements, were part of the following groups: High dose of psilocybin with CI priming (HD CI) (*N* = 30); High dose with AB priming (HD AB) (*N* = 20); Low dose with CI priming (LD CI) (*N* = 25); Low dose with AB priming (LD AB) (*N* = 25). Baseline demographic and experiential variables for the full sample and each experimental group are summarized in Table 1. Age is reported as mean ± standard deviation, whereas baseline depression, anxiety, stress, previous psychedelic experience, meditation practice, and adverse experiences are reported as median and interquartile range due to their non-normal distributions. The number of participants who completed follow up measures was reduced to 93 and were part of the following groups: High dose of psilocybin with CI priming (HD CI) (*N* = 26); High dose with AB priming (HD AB) (*N* = 19); Low dose with CI priming (LD CI) (*N* = 23); Low dose with AB priming (LD AB) (*N* = 25). High doses comprised 3 g of dry *Psilocybe* mushrooms and low doses comprised 1 g of the same material.

**Table 1:** Baseline demographic and experiential characteristics of the participants.

Variable	All	HD	HD CI	HD AB	LD	LD CI	LD AB
Age (Mean ± SD)	37.28 ± 7.78	35.69 ± 6.42	35.65 ± 6.51	35.74 ± 6.47	38.77 ± 8.67	38.61 ± 9.86	38.92 ± 7.63
Depression^†^	1.00 [0.00, 4.00]	1.00 [0.00, 2.00]	1.00 [0.00, 3.00]	1.00 [0.50, 1.50]	2.00 [0.00, 4.25]	2.00 [0.00, 3.00]	2.00 [0.00, 5.00]
Anxiety^†^	1.00 [0.00, 3.00]	1.00 [0.00, 2.00]	1.00 [1.00, 3.00]	1.00 [0.00, 1.00]	1.00 [0.00, 3.00]	1.00 [0.00, 2.50]	1.00 [0.00, 3.00]
Stress^†^	3.00 [1.00, 6.00]	2.00 [1.00, 5.00]	3.50 [1.00, 7.75]	1.00 [1.00, 2.50]	3.00 [2.00, 6.00]	4.00 [1.50, 6.00]	3.00 [2.00, 6.00]
Previous psychedelic experience^†^	1.00 [0.00, 4.00]	1.00 [0.00, 5.00]	1.00 [0.00, 5.00]	1.00 [0.00, 3.50]	0.00 [0.00, 3.00]	0.00 [0.00, 2.00]	1.00 [0.00, 4.00]
Meditation (hours/week)^†^	0.00 [0.00, 3.00]	0.50 [0.00, 3.00]	0.00 [0.00, 3.00]	1.00 [0.00, 4.00]	0.00 [0.00, 3.00]	0.00 [0.00, 4.00]	0.00 [0.00, 3.00]
Adverse experiences^†^	0.00 [0.00, 0.00]	0.00 [0.00, 0.00]	0.00 [0.00, 0.00]	0.00 [0.00, 0.00]	0.00 [0.00, 0.00]	0.00 [0.00, 0.00]	0.00 [0.00, 0.00]

Age is reported as mean ± SD. All other variables are reported as ^†^median [interquartile range]. No baseline differences between groups survived false discovery rate (FDR) correction.

Psilocybin experiences occurred in a variety of settings, encompassing both indoor and outdoor environments. Outdoor settings, particularly those immersed in natural surroundings with grass, trees, and open spaces, were predominantly favored for these experiences. Facilitators administered the doses by grinding the dry mushrooms and mixing the resulting powder with fruit juice, serving one cup per participant. The amount of powder per cup was weighed to ensure all doses were equal. From the moment of ingestion until 5 h after, participants only interacted with facilitators and their teams, unless they required special attention by the professionals in the research team. Facilitators provided recorded and live music, aromatics, and intervened if deemed necessary by accompanying participants during difficult parts of the experience.

### 2.5 Psychometric, wellbeing and experience scales and questionnaires

Participants completed a series of scales and questionnaires at different time points during the experiment. One week before and after psilocybin intake, participants completed two sets of questionnaires online over 2 consecutive days. Set 1 included Spanish versions of various scales, such as the Big Five personality test (John and Srivastava, 1999), (STAI trait) (Spielberger, 2010), and Tellegen absorption scale (TAS) (Tellegen and Atkinson, 1974). Set 2 included additional scales, focusing on measures of compassion and contemplative abilities such as Compassion Engagement and Action Scales (CEAS) measuring compassion to others, from others and for self (Gilbert et al., 2017), and the Experiences Questionnaire (EQ), Decentering subscale (Fresco et al., 2007), among others.

On the day of psilocybin intake, participants completed pre-experience and post-experience questionnaires, assessing their subjective states and experiences with the drug. The pre-experience set included the Spanish versions of: State Anxiety Inventory (STAI state) (Spielberger, 2010), and MAIA scale (Mehling et al., 2012). The post-experience set included the Spanish versions of: State Anxiety Inventory (STAI state) (Spielberger, 2010), 21 items presented in the form of a visual analog scale (VAS) adapted from Carhart-Harris et al. (2016) to determine the intensity of the acute effects, AWE (Yaden et al., 2019), the mystical experience questionnaire (MEQ-30), MEQ Setting, MEQ Social factors, MEQ Fusion (Barrett et al., 2015), 5D altered states of consciousness scale (5D-ASC) (Studerus et al., 2010) and MAIA (Mehling et al., 2012).

Follow-up measures were conducted virtually at 30, 60, and 180 days after the ceremony, including the same sets of questionnaires (Set 1 & 2) with the addition of an Events of Life Scale (Casullo, 2004). Due to follow-up measure dropouts, the self-reported questionnaire dataset was reduced to 93 participants.

The complete list of measures are provided in Supplementary Material. Each questionnaire was scored as described in the corresponding citation. Those that presented unbalanced experimental grouping at baseline were excluded from the analysis.

### 2.6 fMRI data acquisition

Two BOLD-weighted fMRI scans were acquired 2 to 4 days before and after psilocybin intake: resting state (RS) and compassionate imagery task (CIT). Scans were collected using a Philips Ingenia 3T scanner equipped with a 16-channel phased array head coil with direct digital sampling. Functional images were acquired using a GRE-EPI sequence (TR/TE = 3000/30 ms, field of view = 240 mm, acquisition matrix = 80 × 80, SENSE factor = 3, flip angle = 90°). Fifty-eight oblique axial slices were acquired in an interleaved fashion, each 3.0 mm thick with no slice gap (3.0 mm isotropic voxels). Each run lasted 5 min. During the RS scan, participants were instructed to remain still and awake with no explicit instructions regarding the content of their thoughts. During the CIT scan, participants received a brief instruction and were asked to construct a compassionate mental image. Data acquisition began immediately following this instruction.

### 2.7 fMRI pre-processing

Preprocessing was performed using FSL and in-house MATLAB scripts. Functional images were first subjected to spatial smoothing using a 5 mm FWHM Gaussian kernel, band-pass filtering (0.01–0.1 Hz), brain extraction (BET), and normalization to standard space (2 mm MNI template). Nuisance signal regression was then applied by removing displacement parameters, mean signals from white matter and ventricular regions, and their first temporal derivatives from the voxel-wise BOLD time series. Global signal regression was not performed. The residual time series were retained for further analysis. Subsequently, motion-related artifacts were addressed using volume censoring (scrubbing). Participants were excluded if more than 20% of volumes exceeded a framewise displacement threshold of 0.5 mm (Power et al., 2019).

For region-based analyses, voxel-wise time series were averaged within each region of interest (ROI) defined by the Automated Anatomical Labeling (AAL) atlas, considering 90 cortical and subcortical non-cerebellar regions (Tzourio-Mazoyer et al., 2002). In addition, resting-state network (RSN) analyses were performed by averaging signals within eight canonical RSNs defined by Beckmann et al. (2005), using publicly available masks (http://www.fmrib.ox.ac.uk/analysis/royalsoc8).

### 2.8 Multivariate machine learning classifiers

Functional connectivity (FC) matrices were computed for each participant and condition by calculating Pearson correlation coefficients between ROI time series. In parallel, connectivity between RSNs (RSN connectivity, RSNC) was also computed and used as a lower-dimensional feature representation. These matrices served as input features for classification analyses.

Random forest classifiers (Breiman, 2001) were trained to distinguish between experimental conditions (pre vs. post, RS vs. CI, and between-group comparisons) using a five-fold cross-validation procedure. Models were implemented using scikit-learn (Abraham et al., 2014), with 1,000 decision trees and a feature subset size equal to the square root of the total number of features. Gini impurity was used as the split criterion, and trees were grown to full depth without pruning. Statistical significance of classifier performance was assessed using permutation testing. Specifically, 1,000 classifiers were trained on data with randomly shuffled labels, and empirical *p*-values were calculated as the proportion of permutation-based accuracies exceeding the accuracy obtained with true labels. Performance was quantified using the area under the receiver operating characteristic curve (AUC), with significance set at *p* < 0.05. The same permutation framework was used to assess feature importance by comparing values obtained from true-label and shuffled-label classifiers.

Three main classification analyses were performed. First, we trained classifiers to distinguish between resting state (RS) and compassionate imagery task (CIT) conditions using pre-intake data from all participants (*N* = 105), in order to capture task-related differences in functional connectivity. Second, we assessed whether changes in RSN functional connectivity associated with the CIT condition (ΔCIT = CIT_POST_-CIT_PRE_) could distinguish between priming conditions (compassion imagery vs. attention to breathing) within each dose group (HD and LD). Thus, classification in this case was performed on subject-level change scores rather than absolute connectivity values.

### 2.9 Statistical analysis

Statistical analyses were conducted using the *statsmodels* Python library (https://www.statsmodels.org/stable/index.html). Normality was assessed using skewness and kurtosis values, which were all within the recommended parameters of 2 for skewness and 7 for kurtosis (West et al., 1996). Participants with significant missing data for measures at multiple timepoints were excluded from relevant analyses.

Instead of conducting a full factorial omnibus test, we performed a directional planned contrast comparing both meditation groups specifically under the high-dose condition, to test whether the effects of the high dose of psilocybin depend on the specific meditation priming. For this purpose, non-paired *t*-tests were used to compare self-reported questionnaire scores between groups and within groups between time points, determining statistical significance at *p* < 0.05, FDR corrections were performed and differences sustained after correction reported. Specifically, this analysis was performed to compare the dose, meditation priming, and the meditation priming for the high dose condition.

Correlational analyses between fMRI features and the scores of questionnaires were performed using Pearson's linear correlation coefficient and reported significant at *p* < 0.05 and at least a medium effect size for the correlation coefficient (|*R*| > 0.3). Brain-behavior correlations were restricted to questionnaire dimensions showing significant longitudinal effects. Correlations were performed using ΔCIT connectivity changes within the high-dose conditions only, as this was the only comparison yielding significant classification performance. Feature importance values derived from the classifiers were not used as selection criteria for individual connectivity pairs, since they do not constitute univariate statistical effects.

## 3 Results

### 3.1 Experimental protocol overview

The schedule for the measurements conducted before, during and after the psilocybin intake day are summarized in Figure 1. One week prior to the psilocybin intake day, participants completed questionnaires online. During the preceding week, they also underwent fMRI scans which consisted of 5 min of rest alternated with 5 min performing a compassionate imagery task. On the day of psilocybin intake, groups of 5 to 10 participants arrived at the location at 8 am and completed a series of assessments, including questionnaires and recorded interviews. Following these measurements, the participants underwent priming with either short Compassionate Imagery (CI) or Attention to Breathing (AB), each lasting approximately 20 min, followed by another round of individual interviews. During the following 5 h, a facilitator administered psilocybin as previously arranged with the group of participants, without intervention nor interaction with the researchers. Subsequently, participants completed questionnaires to assess the experience. During the following days, the subjects took part in another fMRI recording session with identical instructions and parameters as the one completed before dosing. Participants also completed scales online 7, 30, 90 and 180 days after the experiment. Besides the measurements summarized in Figure 1, we also acquired other data sources (e.g., actigraphy, pre and post dosing EEG, blood and saliva samples) that will be analyzed and reported in future publications.

Baseline demographic and experiential characteristics are summarized in Table 1. Overall, participants were 37.3 ± 7.8 years old and exhibited low baseline levels of depression, anxiety, and stress. Previous psychedelic experience and meditation practice showed considerable interindividual variability, reflecting the heterogeneity inherent to the naturalistic design. Although some nominal differences were observed across groups, none survived false discovery rate (FDR) correction.

### 3.2 Acute effects during the psychedelic experience

**Figure 2:**
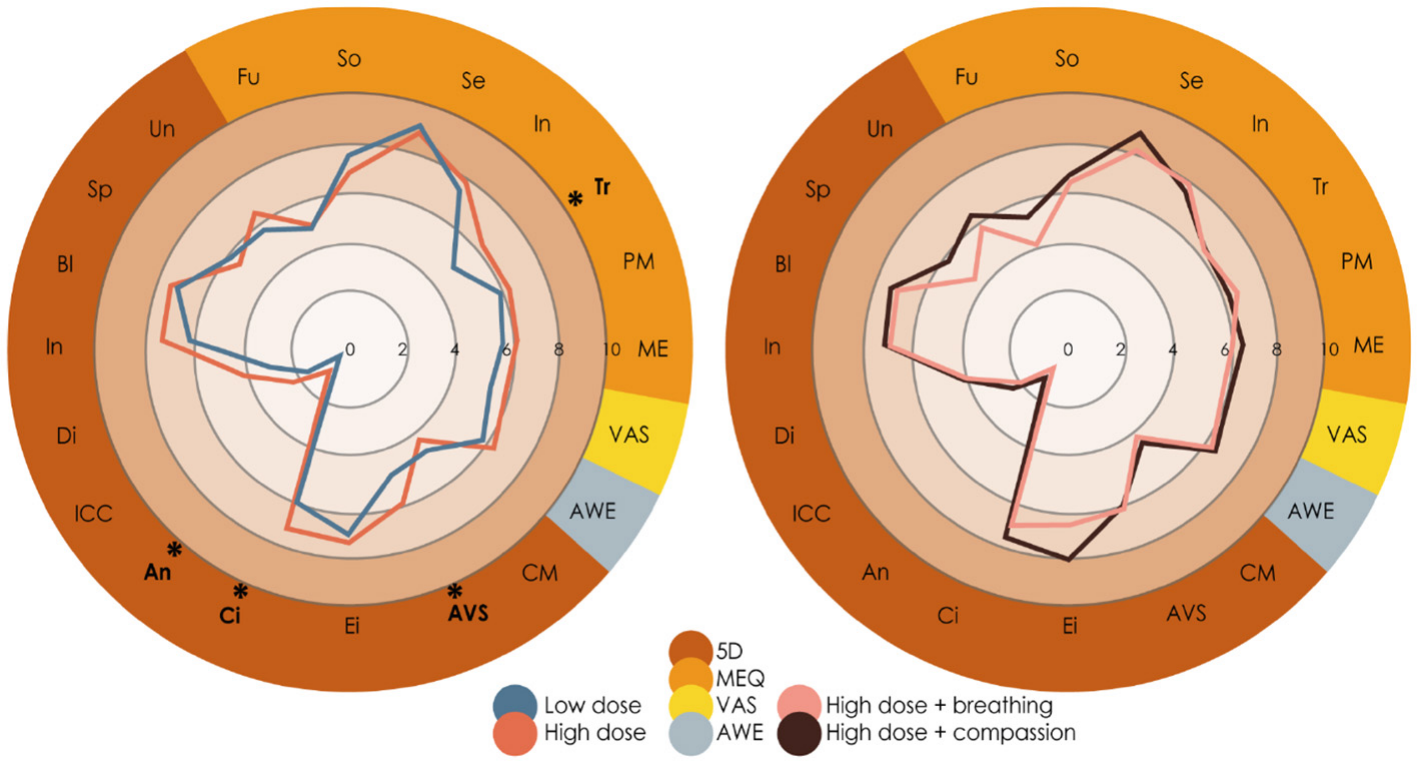
Acute subjective effects of psilocybin are largely similar across dose and priming conditions. Radar plots summarize post-experience questionnaire scores across multiple dimensions derived from the Mystical Experience Questionnaire (MEQ), Visual Analog Scales (VAS), the 5D Altered States of Consciousness scale (5D-ASC), and Awe. **(Left)** Comparison between high-dose (HD) and low-dose (LD) psilocybin conditions. **(Right)** Comparison between compassionate imagery (CI) and attention-to-breathing (AB) priming in the high-dose condition. Values were normalized to a common 0–10 scale for visualization. Asterisks indicate dimensions showing nominally significant differences (*p < 0.05, uncorrected). The observed differences corresponded to small-to-moderate effect sizes (Cohen's *d* = 0.40–0.61). Overall, profiles were highly similar across conditions. However, these results should not be interpreted as evidence of successful blinding, as participants' beliefs regarding condition assignment were not formally assessed.

The radar plots in Figure 2 show the outcome of questionnaires related to the subjective experience elicited by psilocybin, including subscales from the MEQ Mystical Experience Questionnaire) (Barrett et al., 2015), VAS (Visual Analog Scales) [adapted from (Carhart-Harris et al. 2016)], 5D Altered States of Consciousness (5D-ASC) (Studerus et al., 2010), and Awe (Yaden et al., 2019). The left panel compares HD vs. LD, while the right panel compares HD CI vs. HD AB. In the HD vs. LD comparison, significant differences (uncorrected) were observed in four dimensions, including MEQ Transcendence (*d* = 0.61), 5D audiovisual synesthesia (*d* = 0.41), 5D anxiety (*d* = 0.43), and 5D complex imagery (*d* = 0.40). However, these accounted for only a small subset of the 20 dimensions tested, indicating that the two doses elicited broadly similar acute subjective experiences. No significant differences were found between the HD CI and HD AB groups, suggesting that the low-dose active control and the priming conditions were effective in minimizing potential unblinding due to subjective effects.

### 3.3 Effects of psilocybin and priming on questionnaires

Participants responded to a series of questionnaires 7 days before the psilocybin intake day, and 7, 30, 90 and 180 days after that event. For each questionnaire subscale, we performed the following comparisons: HD vs. LD, and HD CI vs. HD AB. The latter comparison aims to capture the synergistic effects of priming and a high dose of psilocybin. Of the 100 participants completing post-intake assessments, 93 completed follow-up measures. Attrition across groups was limited: HD CI (30 → 26), HD AB (20 → 19), LD CI (25 → 23), and LD AB (25 → 25). We performed comparisons between groups for each time point, as well as within-group comparisons between different time points. We excluded subscales (compassion toward others, CEAS toward, and from others CEASFrom), which presented significant differences at baseline from these analyses, indicating that the self-reports made by the different groups of participants were not matched for that dimension (see Supplementary Figure S1). Also 2 of the 3 dimensions of the Compassion Fears scales were unbalanced at baseline which drove us to disregard this measure as well (Supplementary Figure S2).

**Figure 3:**
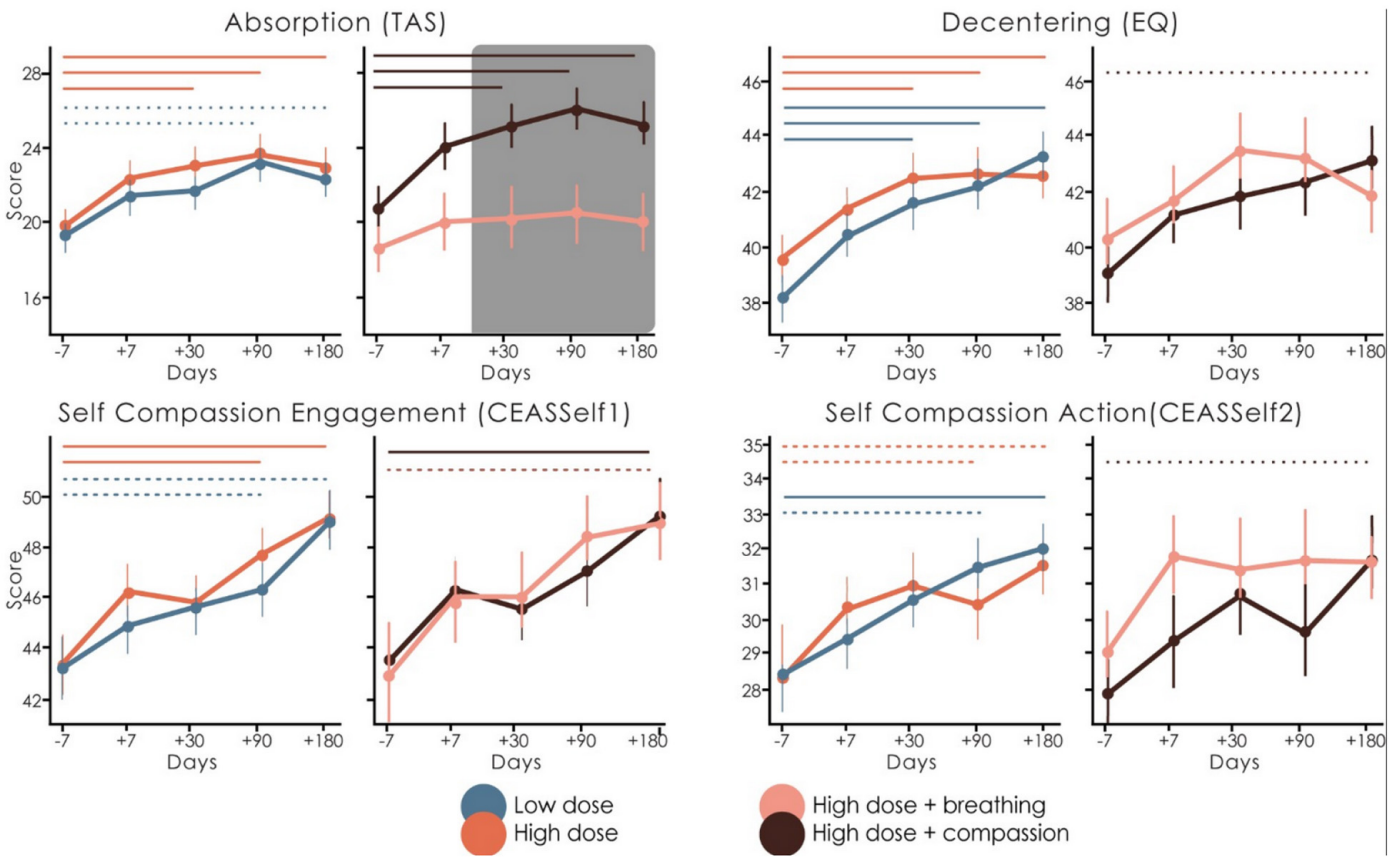
Longitudinal effects of psilocybin dose and contemplative priming on absorption, decentering, and self-compassion. Each subplot summarizes questionnaire scores across multiple time points before and after the psilocybin intake day. Left panels compare low-dose (LD) and high-dose (HD) psilocybin conditions, while right panels compare compassionate imagery (CI) and attention-to-breathing (AB) priming within the high-dose condition. Shaded gray backgrounds indicate time points showing significant between-group differences (*p* < 0.05, FDR-corrected). Colored brackets denote significant within-group changes between time points for the corresponding group (*p* < 0.05), with solid brackets indicating effects surviving FDR correction. Absorption (TAS) showed a sustained increase in the HD CI group relative to HD AB, consistent with a synergistic effect of psilocybin dose and compassionate imagery, whereas decentering (EQ) and self-compassion measures primarily exhibited within-group longitudinal increases.

Figure 3 summarizes the longitudinal effects observed across questionnaire measures. The most robust finding was a sustained increase in absorption (TAS), particularly in the HD CI condition, which also showed significantly higher scores than HD AB at multiple follow-up time points. Participants in the HD condition showed significant FDR-corrected increases relative to baseline at +30 (*d* = 0.48), +90 (*d* = 0.57), and +180 (*d* = 0.48) days. In contrast, the LD condition exhibited only a tendency toward increased absorption at +90 and +180 days, which did not survive correction for multiple comparisons. In the priming comparison, TAS scores were significantly higher in the HD CI group than in the HD AB group at +30 (*d* = 0.72), +90 (*d* = 0.82), and +180 (*d* = 0.76) days (all FDR-corrected). Within-group analyses further revealed that the HD CI group presented robust increases from baseline to these same time points (all FDR-corrected), while no significant longitudinal effects were observed for HD AB.

On the Experiences Questionnaire (EQ)—Decentering subscale, both HD and LD conditions showed significant FDR-corrected increases at multiple time points after psilocybin intake (*d* = 0.48–0.83). For the priming comparison, we observed a trend toward greater increases in the HD CI group relative to baseline, with the effect approaching significance at +180 days, whereas no such effect was observed for HD AB.

On the Self compassion scales (CEASSelf1 & CEASSelf2), we observed differential within-group effects for engagement and action over time. For CEASSelf1 (engagement), the HD group showed significant FDR-corrected increases relative to baseline at +90 (*d* = 0.56) and +180 (*d* = 0.75) days. The LD group displayed similar tendencies at the same time points, though these did not survive correction. Within the priming conditions, the HD CI group exhibited a significant increase at +180 (*d* = 0.76) days, while HD AB only showed a non-significant tendency. For CEASSelf2 (action), both HD and LD conditions displayed tendencies toward increases over time, with only the LD group at +180 (*d* = 0.55) days surviving FDR correction. In the priming comparison, the HD CI group presented a trend toward increased action at +180 days, though this effect did not reach FDR significance. It is important to note that these findings reflect within-group temporal changes and do not imply statistically significant between-group differences. While the observed patterns varied across groups, the current results do not provide sufficient evidence to conclude that one condition or intervention was more effective than another. Nevertheless, reporting these trends is valuable for generating hypotheses and identifying potentially meaningful directions for future research, particularly given the exploratory nature of the study and the novelty of combining psilocybin with compassion-based interventions.

Overall, these results indicate that psilocybin intake, particularly at high doses and when combined with compassionate imagery, was associated with sustained increases in absorption, and showed trends toward enhanced decentering and self-compassion engagement up to 6 months after the experience, warranting further investigation.

### 3.4 fMRI-based classification of experimental conditions

Based on previous neuroimaging findings, we assessed whether the experimental conditions were associated with differences in functional connectivity at the network level using a multivariate machine learning approach. Random forest classifiers were trained to perform two main classification analyses based on RSN connectivity (RSNC). First, we classified resting state (RS) vs. compassionate imagery task (CIT) functional connectivity using pre-intake data from all participants (*N* = 105), in order to capture intrinsic differences between conditions. Second, we assessed whether changes in CIT RSN functional connectivity following psilocybin intake (ΔCIT = CIT_POST_-CIT_PRE_) could distinguish between priming conditions (CI vs. AB) within each dose group (HD and LD). In this case, classification was performed on subject-level change scores rather than absolute connectivity values. We evaluated classifier performance separately for the HD and LD groups and observed significant classification only in the HD condition (Supplementary Figure S3).

Each machine learning classifier was trained using the FC between an established set of RSN comprising primary and extrastriate visual networks (VI and ES), an auditory network (AU), the sensorimotor cortex (SM), the default mode network (DM), the fronto-parietal executive control network (EC), and the right/left dorsal attention network (DR and DL) (Beckmann et al., 2005). For each evaluation, 1,000 random forest classifiers were constructed, both with real data and scrambled labels for the calculation of *p*-values associated with the classifier accuracy and the feature importance reflecting the relevance of each pairwise FC for the classifier, as described in the Methods section. The accuracies were determined using the Area Under the Curve (AUC) of the Receiver Operating Characteristic (ROC) curve (< AUC>) of the 1,000 repetitions.

**Figure 4:**
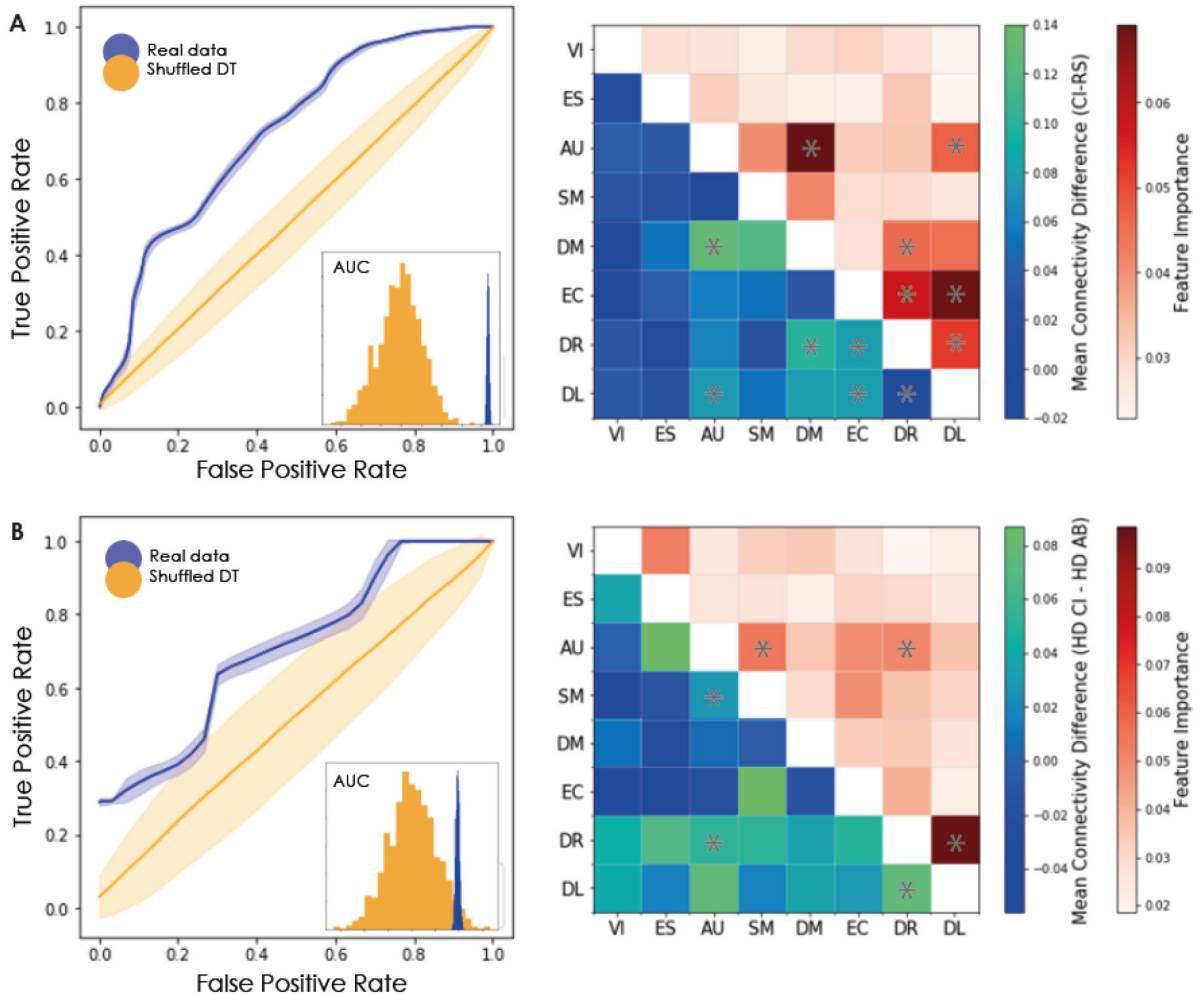
Functional connectivity–based classification of task and priming conditions. Random forest classifiers were trained on resting-state network (RSN) connectivity to discriminate between experimental conditions. **(A)** Classification between resting state (RS) and compassionate imagery task (CIT) scans across participants. **(B)** Classification between compassionate imagery (CI) and attention-to-breathing (AB) priming within the high-dose (HD) psilocybin condition, based on changes in CIT connectivity before vs. after intake. Left panels show receiver operating characteristic (ROC) curves for classifiers trained on real data (blue) and on shuffled labels (orange). Insets show histograms of AUC values obtained from real data (blue) and 1000 label permutations (orange), with each bin indicating the frequency of permutation-derived AUCs. Right panels show connectivity matrices summarizing classifier results: entries above the diagonal represent mean feature importance for each RSN–RSN connection, while entries below the diagonal indicate mean connectivity differences between conditions (RS—CIT in A; ΔHDCI—ΔHDAB in **B**). Asterisks denote connections showing significant effects (*p* < 0.05, permutation-based). In Panel B, significant classification performance was observed only in the high-dose condition; classification in the low-dose condition did not exceed chance levels (see Supplementary Figure S3).

As shown in Figure 4, the classifier successfully distinguished between both sets of experimental conditions. In the case of RS vs. CI, this classification was predominantly based on increased FC values between the DR and DL, and between these two networks and EC, DM and AU (< AUC> = 0.716 ± 0.008; *p* < 0.001) (Figure 4A). In the comparison of connectivity differences before and after intake in the CI task (ΔCIT) between the AB and CI priming, we recovered a significant positive classification (< AUC> = 0.696 ± 0.008; *p* < 0.05) for the high dose conditions (Figure 4B). We found significant increases in the coupling between the DR and DL networks, and between AU and both SM and DR. However, the same comparison for the low dose groups did not result in accurate classification (< AUC> = 0.49 ± 0.01; *p* > 0.5), illustrated in Supplementary Figure S2. Given that significant classification performance was observed only for ΔCIT connectivity changes in the high-dose groups, subsequent brain-behaviour analyses were restricted to this contrast.

### 3.5 Correlations between fMRI and self-reported questionnaires

**Figure 5:**
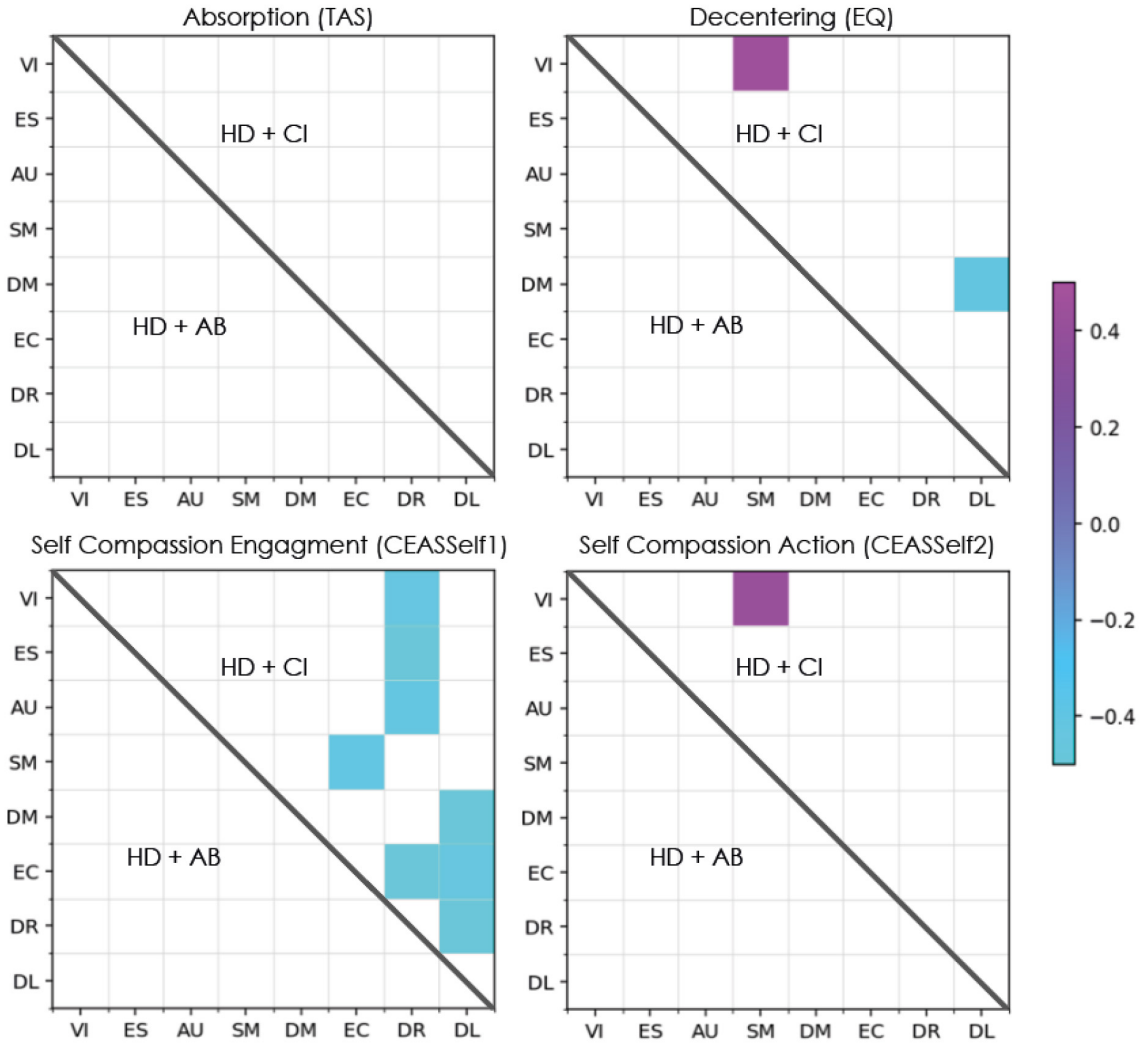
Associations between changes in functional connectivity during compassionate imagery and self-reported measures. Each panel shows correlations between changes in resting-state network (RSN) functional connectivity during the compassionate imagery task (ΔCIT = CITPOST–CITPRE) and questionnaire scores for absorption (TAS), decentering (EQ), self-compassion engagement (CEASSelf1), and self-compassion action (CEASSelf2). Matrix entries above the diagonal correspond to the high-dose compassionate imagery group (HD CI), while entries below the diagonal correspond to the high-dose attention-to-breathing group (HD AB). Only correlations exceeding |*R*| > 0.3 and *p* < 0.05 are shown. Associations were predominantly observed in the HD CI group, whereas few or no significant correlations were detected in the HD AB group, indicating that relationships between connectivity changes and self-reported measures were specific to the compassionate imagery condition under high-dose psilocybin.

Afterwards, we investigated the correlations between changes in RSN FC and the dimensions of the self-reported questionnaires (measured on the same time-point as the fMRI data) for which we previously found a significant effect of dose, or an effect associated with the combination of dose and priming (Figure 3). The results of this analysis are shown in Figure 5. Importantly, all correlations were computed on change scores in functional connectivity during the compassionate imagery (CI) task (ΔFC = POST CI–PRE CI) rather than on absolute FC measures. Thus, the associations below should be interpreted as linking individual variability in CI-evoked connectivity change to questionnaire scores. As mentioned above, these analyses were only performed for the HD groups due to their significant classification.

TAS showed no significant correlations in either dose condition. By contrast, all other scales exhibited significant associations only in the HD CI group. Specifically, EQ (Decentering) and CEASSelf2 (action) showed positive correlations with connectivity between visual cortex and sensorimotor (VI-SM) networks, indicating that larger increases in decentering and self-compassionate action were associated with stronger VI-SM integration across sessions. In turn, CEASSelf1 (engagement) displayed predominantly negative correlations, including reduced coupling between the dorsal attention networks (DL, DR) and the rest of the RSNs, and a negative EC-SM association.

## 4 Discussion

Although both psychedelic intake and compassion-based practices have been individually associated with increased psychological flexibility and adaptive emotional processing, their combined influence within a shared experimental framework remains relatively unexplored. In this study, we investigated whether psilocybin-related effects on key markers of wellbeing, including decentering, self-compassion, and absorption, would vary as a function of compassion imagery priming. We employed self-report questionnaires assessing stable traits and psychological processes, alongside fMRI recordings acquired before and after psilocybin intake, with participants undergoing either compassion imagery or attention-to-breathing priming. Given the novelty of this approach, our analyses were exploratory in nature.

Since the contemporary renewed interest in psychedelic-assisted psychotherapy, obtaining a reliable control for placebo effects has remained a persisting challenge, given the difficulties of masking the subjective aspects of the experience (Luoma et al., 2020; Nayak et al., 2023). To address this issue, we implemented a low-dose condition (1 g of dried mushrooms) as an active comparator to the high dose (3 g), within a naturalistic setting in which participants shared the experience in groups. When comparing self-reported subjective effects during the acute phase, we observed only limited differences across a few dimensions between high and low doses, and no significant differences between HD CI and HD AB (Figure 2). While this pattern suggests that subjective experience measures did not strongly differentiate between conditions, it does not constitute a direct assessment of blinding, as participants' beliefs about the received dose were not explicitly measured. Nevertheless, the relative similarity in reported experiences may have reduced overt cues about condition assignment, potentially attenuating expectancy-related differences between groups. In this context, the lack of strong subjective differentiation may have contributed to smaller observed between-group effects. It is well-documented that outcomes in psychedelic research often have an expectancy component, where participants‘ beliefs can influence results (Olson et al., 2020; Muthukumaraswamy et al., 2021; Aday et al., 2022; Szigeti and Heifets, 2024). Accordingly, if a substantial proportion of participants believed they were receiving a high dose, this could have attenuated differences between experimental conditions, potentially contributing to reduced effect sizes. Finally, expectancy effects cannot be ruled out and should be considered when interpreting the present findings.

An interesting result reflected in our data is the consistent increase in absorption, as measured by the Tellegen Absorption Scale (TAS). The HD group showed robust, FDR-corrected increases at multiple follow-up time points, while the LD group displayed non-significant tendencies in the same direction. This pattern suggests that psilocybin in this context is associated with increased absorption, with higher doses linked to more reliable and sustained effects. Absorption describes the propensity to become fully engaged in perceptual or imaginative experiences (Tellegen and Atkinson, 1974) and has been linked to openness to altered states of consciousness and to contemplative practices such as meditation (Pekala et al., 1985; Radtke and Stam, 1991; Cuevas-Vargas et al., 2023; Yang et al., 2024). In this sense, increased absorption may be viewed as an externally driven contemplative ability (Lifshitz et al., 2019), with the psychedelic state potentiating immersion in inner imagery or sensory experience (Aday et al., 2021). We also observed that the HD CI group showed significantly greater increases in TAS relative to HD AB, both at specific time points and across baseline-to-follow-up comparisons. This pattern suggests that compassion imagery may have influenced the extent of absorption increases observed under high-dose psilocybin. Such outcomes resonate with evidence linking contemplative training to absorption-related capacities (Millière et al., 2018) and highlight the potential of combining psychedelics with structured practices to support the development of contemplative traits. In this context, enhanced absorption may underlie the sustained ability to engage deeply with compassionate imagery and other self-transcendent experiences, bridging acute psychedelic phenomenology and enduring changes in wellbeing (Haijen et al., 2018; Lifshitz et al., 2019; Aday et al., 2021).

Another finding was the consistent increase in decentering, as measured by the Experiences Questionnaire (EQ). Both HD and LD groups showed significant FDR-corrected increases at multiple follow-up time points. A trend toward stronger effects was observed in the HD CI group at 6 months, suggesting that compassionate imagery may modestly potentiate this trait over time. Decentering refers to the metacognitive ability to observe one's thoughts, feelings, and internal experiences as temporary, objective events in the mind rather than as accurate reflections of the self or reality (Fresco et al., 2007). It involves a shift from being immersed in one's inner narrative to adopting a witnessing or observational stance, which enables greater psychological flexibility and emotional regulation. This ability supports adaptive self-reflection and has been linked to cognitive reappraisal, the process of changing the meaning of experiences by re-evaluating emotions toward them (Gross and John, 2003; Bernstein et al., 2015; Kobayashi et al., 2020). Recent work highlights decentering as a key mechanism in contemplative practices, including mindfulness-based interventions and compassion-based training, where it underpins resilience and psychological flexibility (Campo and Yali, 2025). Importantly, emerging evidence suggests that psilocybin may facilitate both self-compassion and decentering by disrupting rigid self-referential processing, loosening habitual patterns of cognitive fusion, and fostering openness to alternative perspectives and modes of self-relatedness (Diehl and Rosenthal, 2024). These shifts are thought to underpin therapeutic change by enhancing psychological flexibility, a core process targeted in various transdiagnostic interventions (Pilecki et al., 2024). Enhancing decentering creates a psychological stance conducive to flexibility, reduced rumination, and prosocial engagement. The trend toward stronger effects in the HD CI group suggests that compassionate priming may contribute to this process, extending the overlap between psychedelic phenomenology and compassionate imagery. By fostering the capacity to observe thoughts and emotions without over-identification, psilocybin combined with compassion imagery may support longer-term changes in psychological flexibility and wellbeing.

Another key finding of this study was an increase in self-compassion over time, particularly in the high-dose condition, with trends toward stronger effects when combined with compassion imagery (Figure 3). To our knowledge, this study provides initial evidence linking measures of self-compassion with psychedelic use. Self-compassion refers to the ability to direct the evolved motivation to care and alleviate suffering inward, fostering a courageous and wise stance toward one's own pain (Gilbert, 2000, 2014). It involves two interrelated components: engagement, the motivation to face and understand suffering in self and others rather than avoiding, denying or suppressing it, and action, the intentional effort to alleviate that suffering through helpful thoughts or behaviors (Gilbert, 2020, 2022; Petrocchi et al., 2024). Rooted in the evolved care motivational system that promotes caring and responses to being cared for, self-compassion activates physiological states of safeness and affiliation that counteract threat responses and regulation (Kim et al., 2020). Research has shown that cultivating self-compassion through CFT is associated with reduced self-criticism, shame, anxiety, and depression, as well as improved emotion regulation and resilience (Gilbert and Procter, 2006; Petrocchi et al., 2024). More recently, Petrocchi et al. (2024) found that CFT produced a large and consistent increase in self-compassion in both clinical and non-clinical populations. There is now growing evidence of an association between self-compassion and a range of health-promoting behaviors, including greater psychological flexibility and resilience (Homan and Sirois, 2017; Eghbali et al., 2022; Tiwari et al., 2020).

The present findings can also be considered in light of the REBUS (Relaxed Beliefs Under Psychedelics) framework (Carhart-Harris and Friston, 2019), which proposes that psychedelics reduce the precision weighting of high-level priors, increasing the flexibility with which beliefs and self-related models are updated. Within this framework, the observed increases in absorption, decentering, and self-compassion may be interpreted as manifestations of greater psychological flexibility and reduced adherence to habitual self-referential patterns. Absorption may reflect an increased capacity to engage with sensory and imaginative experience, while decentering reflects the ability to observe thoughts and emotions without over-identification. Similarly, increased self-compassion may emerge from a temporary loosening of rigid self-evaluative beliefs, allowing the development of more affiliative and caring modes of self-relatedness. Although the present study was not designed to directly test REBUS predictions, these observations are broadly consistent with the hypothesis that psychedelics facilitate adaptive belief updating and cognitive flexibility.

In summary of our self-report findings, we observed sustained increases in absorption, with significantly greater increases in the high-dose compassionate imagery group relative to the high-dose control condition. While increases in decentering and self-compassion were also observed over time within certain groups, these did not yield significant between-group differences and should be interpreted as trends. Together, these patterns suggest that psilocybin, particularly when combined with compassion imagery priming, may support the engagement of care-affiliative motivational processes over time, providing a potential account of sustained effects observed up to 6 months following psilocybin administration.

Our analysis of fMRI recordings successfully differentiated between resting state and compassionate imagery tasks performed within the scanner (Figure 4A), highlighting the recruitment of distinct neural configurations associated with each condition. The classification of these two conditions primarily relied on network-level functional connectivity (RSNC) among the left and right dorsal attention networks (DL and DR), as well as between these networks and the fronto-parietal executive control network (EC), default mode network (DM), and auditory network (AU). The dorsal attention networks (DANs) encompass the dorsal frontal and parietal cortices, specifically the intraparietal sulcus (Beckmann et al., 2005). These networks are implicated in the voluntary orientation and maintenance of attention to a location (Corbetta et al., 2000) and are considered goal-driven attentional systems that utilize internal goals or expectations to guide sensory processing (Corbetta et al., 2008). There is evidence linking the connectivity of these networks with mindfulness and contemplative traits (Parkinson et al., 2019; Froeliger et al., 2012), connecting the functions of the DANs to the sustained attentional demands of contemplative practices. In this context, the observed connectivity patterns during compassionate imagery may reflect increased engagement of attentional control processes. Another key observation is that most RSN connectivities contributing to the distinction between RS and CIT were higher during the imagery condition. Since resting-state networks are often characterized by patterns of functional segregation, this relative increase in connectivity suggests that compassionate imagery may be associated with a more integrated network configuration. Such patterns parallel previous reports of increased network integration during psychedelic states, suggesting a potential overlap in large-scale network dynamics associated with internally oriented or expanded experiential states.

Network-level functional connectivity also enabled the distinction between priming conditions (AB vs. CI) in the high-dose group when considering changes in connectivity during the compassionate imagery task (ΔCIT = CIT_post_-CIT_pre_; Figure 4B). This finding suggests that psilocybin-related changes in CI-evoked connectivity patterns differed as a function of priming condition. As in the RS vs. CIT classification, connectivity within the dorsal attention networks (DR and DL) played a central role, along with connections between the auditory network (AU) and both the sensorimotor network (SM) and DR. The involvement of the SM network may reflect the embodied component of the CI task, as sensorimotor integration contributes to grounding perceptual and interoceptive experience (Shimada, 2022; Ferri et al., 2012). Notably, discriminative connectivity features were predominantly positive, indicating larger changes in connectivity in the HD CI group relative to HD AB. These patterns suggest that compassionate imagery priming may modulate the extent of connectivity changes observed under psilocybin, particularly in networks related to attention and sensorimotor integration. Considering available evidence linking regions of the insula to compassionate states and traits (Kim et al., 2020; Novak et al., 2022), and given that this region is included within the AU network (Damoiseaux et al., 2006), the observed involvement of this network may be consistent with the engagement of processes related to interoception and affective integration during compassionate imagery.

From the perspective of REBUS (Carhart-Harris and Friston, 2019), the observed changes in functional connectivity may also be interpreted as reflecting altered interactions between large-scale brain systems involved in attention, self-referential processing, and embodied cognition. However, because our analyses were conducted at the level of resting-state networks and did not directly assess predictive processing or precision-weighting mechanisms, these interpretations remain speculative and should be considered as potential theoretical links rather than direct tests of the model.

Lastly, we explored the relationship between changes in network-level functional connectivity during the compassion imagery task (ΔCIT) and self-reported measures, focusing on the comparison between the HD CI and HD AB groups to more directly assess differences associated with compassion priming under psilocybin (Figure 4). Notably, no significant correlations emerged in the HD AB group, suggesting that psilocybin combined with attention-to-breath priming may be associated with weaker or less consistent links between brain dynamics and self-reported psychological outcomes. In contrast, multiple significant correlations were observed in the HD CI group, indicating a more consistent relationship between connectivity changes and subjective measures in this condition. A particularly relevant finding concerns the SM, a key hub in task-driven processes and in grounding the inner narrative self (Ferri et al., 2012; Weiss, 2013). Its positive connectivity with EQ and CEASSelf2 suggests that enhanced integration of embodied and perceptual networks may be related to the capacity to disengage from rigid self-referential processes and support compassionate action (Farb et al., 2007; Ferri et al., 2012). In parallel, negative correlations between the DL and the DM with decentering are consistent with the idea that psychological flexibility involves a rebalancing between attentional and self-referential systems (Farb et al., 2007; Christoff et al., 2016). Furthermore, CEASSelf1 was associated with widespread negative correlations involving both dorsal attention networks (DR and DL), suggesting that reduced connectivity between these networks and the rest of the brain may be linked to a more focused, inwardly directed mode of compassionate engagement (Corbetta et al., 2008; Parkinson et al., 2019; Garrison et al., 2015). These results resonate with the CFT model, which distinguishes two complementary components of self-compassion: engagement and action. The engagement aspect (CEASSelf1), characterized by reduced connectivity across dorsal attention and executive-sensorimotor networks, may reflect a shift away from externally oriented, goal-directed processing toward a more open and receptive internal awareness. In contrast, the action component (CEASSelf2) was linked to increased visual-sensorimotor connectivity, consistent with enhanced embodiment and perception-action integration, which may support compassionate responding. Together, these findings highlight the involvement of task-positive networks (SM, DR, DL) and their interaction with self-referential systems in relation to the psychological effects observed under psilocybin combined with compassion imagery. The alignment of these neural patterns with reported changes in decentering and self-compassion points to a potential mechanism through which contemplative and caring capacities may be supported at the network level. The consistent implication of the dorsal attention networks further suggests a potentially relevant role for these systems in supporting the mental states associated with compassion imagery, warranting further investigation in future research. Because the correlation analyses were exploratory and were not corrected for the large number of possible brain-behaviour relationships, the reported associations should be interpreted as hypothesis-generating. Future studies with larger samples and confirmatory designs will be required to establish the robustness and specificity of these relationships.

The results of our study present a series of limitations. While investigating psychedelics in a natural context presents multiple advantages, it also imposes limitations related to lack of control in the administered doses, and to the difficulty of adopting and following a double-blind design. Because participants' beliefs regarding dose assignment and priming condition were not systematically assessed, the effectiveness of blinding remains unknown. We could distinguish between low and high psilocybin doses; however, each dose was administered to a different group of participants, thus preventing us from performing within-group comparisons. This limitation was manifest in some metrics that presented significant differences at baseline, indicating that groups were not perfectly matched in that dimension. Moreover, the lack of significant results in the acute effects measurements could stem from participants lacking an adequate reference value to judge the intensity of the effects. Also, as participants were free to withdraw without justification, reasons for attrition were not systematically collected. Although overall attrition was relatively low, participant loss over the follow-up period may have introduced bias in longitudinal analyses and should be considered when interpreting long-term effects.

Another important limitation concerns the absence of chemical characterization of the mushroom material. Doses were defined according to the weight of dried *Psilocybe* mushrooms rather than quantified psilocybin content. Alkaloid concentrations are known to vary as a function of strain, flush number, fruiting body variability, mushroom part, and storage conditions (Beug and Bigwood, 1982; Gotvaldová et al., 2021; Kurzbaum et al., 2025). As a consequence, participants receiving the same nominal dose may nevertheless have received different amounts of active compounds, increasing inter-individual variability and reducing experimental precision. In the present study, however, the mushroom material was ground and homogenized prior to administration, and facilitators and sourcing procedures remained stable throughout the experiment, likely increasing consistency in strain, cultivation, drying, storage, and handling conditions. Thus, although variability in alkaloid content represents an uncontrolled source of error that likely increased noise and reduced statistical power, it is unlikely to have reversed the intended dose hierarchy, whereby participants in the nominal 3 g condition would have systematically received lower psilocybin exposure than those in the 1 g condition. Although this limitation reflects the reality of naturalistic psilocybin use and therefore contributes to the ecological validity of the study, it constrains the interpretation of dose-related effects and limits direct comparison with studies using standardized pharmaceutical psilocybin preparations.

Due to time constraints, the training in compassion-focused imagery received by the participants was brief at 20 min; therefore, future studies with higher intensity or longer duration compassion-focused training are needed to replicate the findings. An important limitation concerns the lack of random assignment and prospective group balancing. Because participants were enrolled on a rolling basis and selected among available ceremony dates, experimental groups may have differed in variables not assessed or controlled for, including prior psychedelic experience, contemplative practice, motivation for participation, and socioeconomic factors. Although no baseline demographic or experiential differences survived FDR correction, the lack of randomization and prospective group balancing means that residual differences in measured or unmeasured variables may have persisted. These uncontrolled sources of variability limit causal inference and may have contributed to both baseline differences and variability in outcomes. Additionally, fMRI analyses were conducted at the network level, which limits spatial specificity and precludes region-level interpretations. Finally, the non-invasive nature of fMRI recordings should be complemented with the analysis of biological markers, which will be reported in future publications.

Finally, the study was not preregistered. Combined with the large number of questionnaires, connectivity measures, and potential comparisons, this increases the risk of selective interpretation and reinforces the need to view many of the present findings as exploratory. Future preregistered and confirmatory studies will be necessary to establish the robustness and generalizability of these observations.

In conclusion, our study shows that psilocybin, particularly when combined with compassion-focused imagery, is associated with sustained increases in absorption, and suggests trends toward enhanced decentering and self-compassion, psychological processes that may contribute to greater flexibility and wellbeing. Absorption provides the attentional immersion necessary to engage with inner experience, decentering allows individuals to relate more flexibly to thoughts and emotions, and compassion activates the caring system, creating a sense of safeness. Together, these mechanisms may interact in a complementary manner: compassion imagery, particularly in high-dose psilocybin contexts, may function as a contextual priming mechanism that supports key experiential processes. By activating affiliative motivation and soothing physiological systems, compassion-based interventions may facilitate processes such as absorption, decentering, and self-compassion, while supporting greater neural and psychological integration. The specificity of the effects observed in the high-dose compassion imagery (HD CI) group highlights the relevance of emotional safeness and affiliative states as potential modulators of psychedelic experiences. Accordingly, future research could investigate the integrative potential of Compassion-Focused Therapy (CFT) and Compassionate Mind Training (CMT), both as preparatory practices and post-experience frameworks to support sustainable therapeutic and self-transformative outcomes.

## Data Availability

The datasets presented in this study can be found in online repositories. The names of the repository/repositories and accession number(s) can be found below: Fully anonymized data supporting the findings of this study are publicly available via Zenodo at https://doi.org/10.5281/zenodo.18174765.
