# Supplementary material for: Longitudinal neural and psychological changes following psilocybin under compassion imagery priming
Source: Front Hum Neurosci. 2026 Jul 22;20:1874178. doi: 10.3389/fnhum.2026.1874178 (PMC13439500; doi:10.3389/fnhum.2026.1874178)
Supplement: Supplementary file 1 [file Data_Sheet_1.pdf]

Supplementary Material

1 Supplementary Data

Fully anonymized data supporting the findings of this study are publicly available via Zenodo at **https://doi.org/10.5281/zenodo.18174765**.

2 Supplementary Figures and Tables

2.1 Supplementary Figures

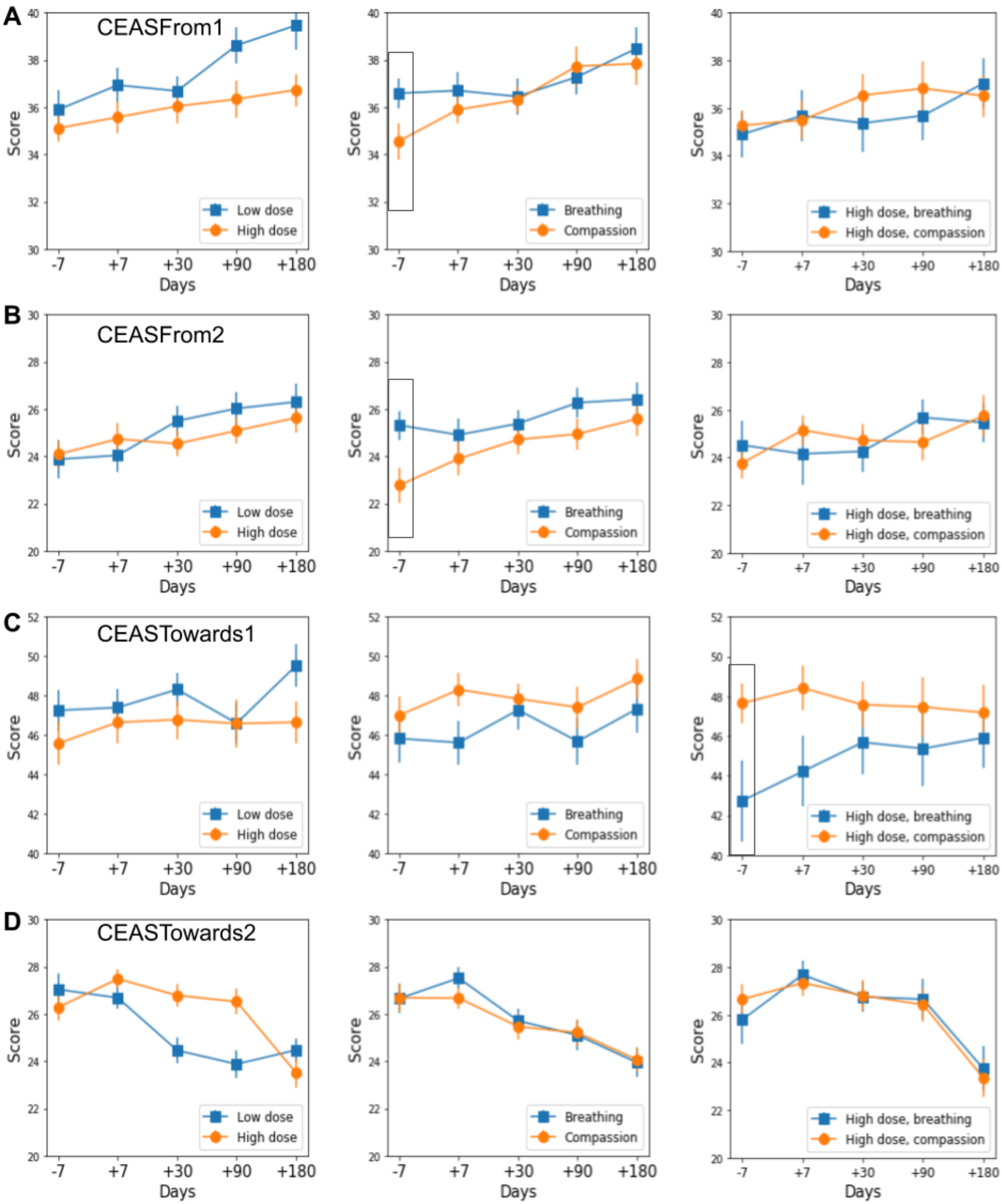

Supplementary Figure S1: *CEAS flows unbalanced at baseline were excluded from further analysis to avoid bias in interpretation.* Each row represents a different CEAS subscale: CEASFrom1 (A), CEASFrom2 (B), CEASTowards1 (C), and CEASTowards2 (D). The column plots display comparisons for HD vs. LD (left), CI vs. AB (middle), and HD CI vs. HD AB (right). Although only the CEASTowards1 (engagement) subscale

showed a significant baseline difference, we opted to exclude the entire flow from group comparisons to maintain analytic consistency and avoid selective reporting. Brackets highlight unbalanced baselines.

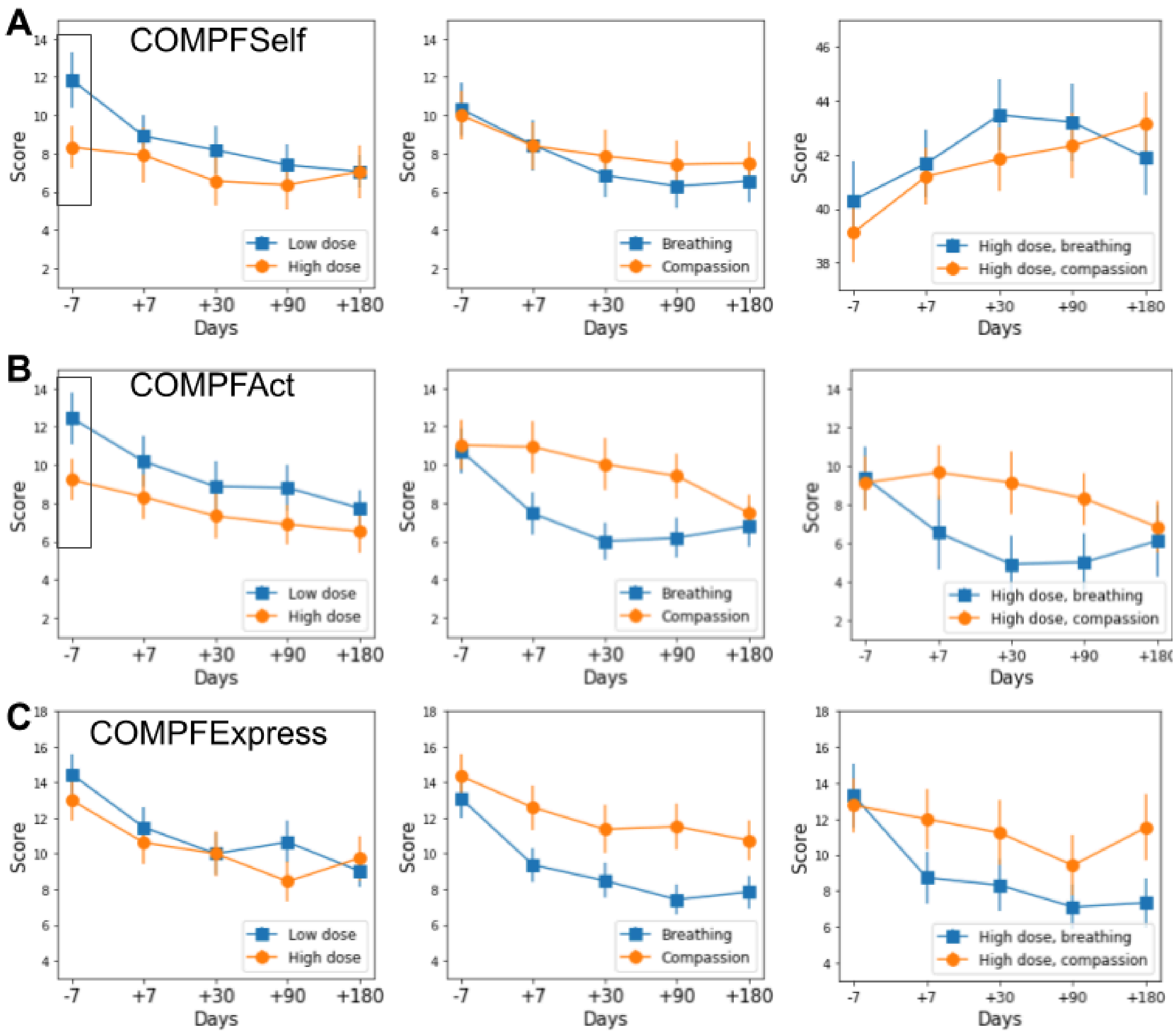

Supplementary Figure S2. *Unbalanced baselines in fears of compassion subscales.* Each row displays a different subscale of the Fears of Compassion scale: COMPFSelf (A), COMPFact (B), and COMPExpress (C). Columns show group comparisons across time for Low vs. High dose (left), Breathing vs. Compassion priming (middle), and High Dose CI vs. High Dose AB (right). Timepoints span from baseline (–7) to +180 days. Two of the three subscales showed significant baseline differences across groups, which led us to exclude this entire metric from further group-level comparisons to avoid biased interpretation and selective reporting. Baseline imbalances are indicated with brackets.

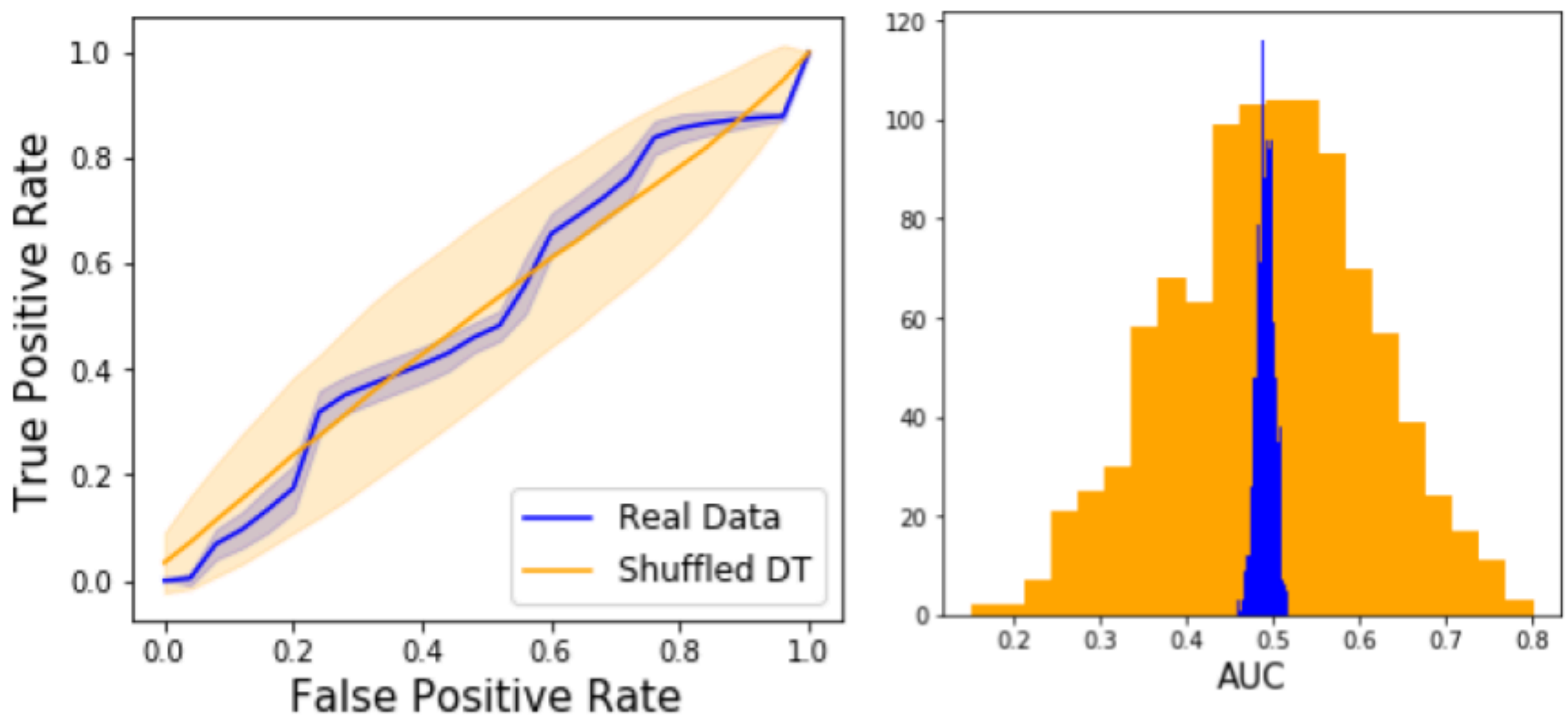

Supplementary Figure S3. *Classification of functional connectivity changes for the low dose condition between the Compassionate Imagery (CI) and Attention to Breath (AB) priming conditions.* The left panel displays the ROC curve using the original data (blue) and the distribution under label permutation (orange). The right panel shows the distribution of AUC values across 1000 random label permutations. Classification performance did not exceed chance level (mean AUC = 0.49 ± 0.01, p = 0.516), indicating the absence of a reliable distinction between the CI and AB groups in the low dose condition.

2.2 Supplementary Tables

**Set 1**		
BFI	Big Five Inventory	Extraversion, Agreeableness, Conscientiousness, Neuroticism, Openness.
STAI	State-Trait Anxiety Inventory	Trait anxiety.
PANAS	Positive And Negative Affect State	Positive affect, Negative affect.
TAS	Tellegen Absorption Scale	Total score.
BIEPS	Escala de Bienestar Psicológico	Acceptance and control, Autonomy, Ties, Projects.
**Set 2**		
CEAS	Compassionate Engagement and Action Scales	To self 1, To self 2, Towards others 1, Towards others 2, From others 1, From others 2.
COMPF	Compassion Fears	Express, Act, Self.
SPS	Social Safeness and Pleasure Scale	Total score.
EQ	Experiences Questionnaire	Decentering
SCS	Self-Compassion Scale	Total score.
CNS	Connectedness to Nature	Total score.
**Pre-experience Set**		
STAI	State-Trait Anxiety Inventory	State anxiety.
MAIA	Multidimensional Assessment of Interoceptive Awareness	Noticing, Not-distracting, Not-worrying, Attention regulation, Emotional awareness, Self-regulation, Body listening, Trusting.
**Post-experience Set**		
STAI	State-Trait Anxiety Inventory	State anxiety.
VAS	Visual Analogue Scale	Total score (VAS).
AWE-S	Awe Experience Scale	Time, Self-loss, Connectedness, Vastness, Physiological, Accommodation. Total Score (AWE)
MEQ	Mystical Experience Questionnaire	Mystical experience (MEQ-ME), Positive mood (MEQ-PM), Transcendence (MEQ-Tr), Ineffability (MEQ-In), Setting (MEQ-Se), Social (MEQ-So), Fusion (MEQ-Fu).
5D-ASC	Five Dimensional Altered States of Consciousness	Unity (5D-Un), Spiritual (5D-Sp), Blissful (5D-Bl), Insightfulness (5D-In), Disembodiment (5D-Di), Impaired consciousness and cognition (5D-ICC), Anxiety (5D-An), Complex imagery (5D-CI), Elementary imagery (5D-EI), Audio-visual synesthesia (5D-AVS), Changed meaning (5D-CM).

Supplementary Material

Set 1		
MAIA	Multidimensional Assessment of Interoceptive Awareness	Noticing, Not-distracting, Not-worrying, Attention regulation, Emotional awareness, Self-regulation, Body listening, Trusting.

Supplementary Table 1. List of all scales and questionnaires corresponding to each set. First Column presents the acronym, second the complete name of the questionnaire and the third lists all the subscales and dimensions within it.
